# Patient Identification for Serious Illness Conversations: A Scoping Review

**DOI:** 10.3390/ijerph19074162

**Published:** 2022-03-31

**Authors:** Rebecca Baxter, Erik K. Fromme, Anna Sandgren

**Affiliations:** 1 Center for Collaborative Palliative Care, Department of Health and Caring Sciences, Linnaeus University, 35195 Växjö, Sweden; anna.sandgren@lnu.se; 2 Ariadne Labs, Boston, MA 02215, USA; efromme@ariadnelabs.org; 3 Harvard Medical School, Boston, MA 02215, USA

**Keywords:** advance care planning, end of life, palliative care, patient identification systems, review, scoping review, serious illness care program, serious illness communication, serious illness conversations

## Abstract

Serious illness conversations aim to align medical care and treatment with patients’ values, goals, priorities, and preferences. Timely and accurate identification of patients for serious illness conversations is essential; however, existent methods for patient identification in different settings and population groups have not been compared and contrasted. This study aimed to examine the current literature regarding patient identification for serious illness conversations within the context of the Serious Illness Care Program and/or the Serious Illness Conversation Guide. A scoping review was conducted using the Joanna Briggs Institute guidelines. A comprehensive search was undertaken in four databases for literature published between January 2014 and September 2021. In total, 39 articles met the criteria for inclusion. This review found that patients were primarily identified for serious illness conversations using clinical/diagnostic triggers, the ’surprise question’, or a combination of methods. A diverse assortment of clinicians and non-clinical resources were described in the identification process, including physicians, nurses, allied health staff, administrative staff, and automated algorithms. Facilitators and barriers to patient identification are elucidated. Future research should test the efficacy of adapted identification methods and explore how clinicians inform judgements surrounding patient identification.

## 1. Introduction

Conversations in serious illness are held to understand and support patients’ values, goals, priorities, and preferences in relation to their health and medical care [1]. Kelley and Bollens-Lund [2] define the term ‘serious illness’ as ‘a health condition that carries a high risk of mortality and either negatively impacts a person’s daily function or quality of life, or excessively strains their caregivers’ (p. S-8). Serious illness conversations have been associated with improved patient outcomes, such as reduced anxiety and suffering, in addition to improved quality of life and satisfaction [3,4,5]. Although scholars recommend having such conversations when patients are relatively stable, all too often eligible patients are not identified until late in the illness process [6,7]. To ensure patients and their families receive care that is concordant with their values, goals, priorities, and preferences, evidence-based approaches are required to identify eligible patients for serious illness conversations in a timely manner.

The Serious Illness Care Program (SICP), developed by Ariadne Labs, aims to equip clinicians with the knowledge and skills to undertake more, better, and earlier serious illness conversations [1,8]. This multicomponent program is comprised of patient identification, clinician training, workflow development, medical record documentation templates, clinician reminders, and evaluation/improvement strategies [9]. The Serious Illness Conversation Guide (SICG) acts as a framework to support discussions between clinicians, patients, and their families about their illness understanding, information preferences, prognosis, key topics (i.e., goals, fears, worries, critical abilities, family involvement, etc.), and clinician recommendations. Serious illness conversations are guided by a person-centered approach in that they provide structure for clinicians to find out what is important to the patient and use this to inform values-based shared decision making and goal-concordant care [10]. This focus on listening and discovering what matters to the patient (physically, psychologically, existentially, relationally) is part of what distinguishes the SICP and SICG from other interventions or conversations in the complex care continuum [10].

While the adaptability of the program and the guide enhances the potential for implementation in a variety of clinical practice arenas (i.e., palliative care, primary care, inpatient/outpatient care, etc.), differences have emerged in how patients and population groups are identified [11]. Bernacki and colleagues [1,6] highlight the importance of developing specific criteria to ‘trigger’ timely identification of eligible patients for serious illness conversations. The ‘surprise question’ (SQ) was the primary method by which patients were identified in the original SICP implementation study [1,6]. This comprised of a single question, ‘would you be surprised if this patient died in the next year?’; where a ‘no’ response identified patients who might benefit from a serious illness conversation. The clinician made the final decision about whether to offer a conversation. However, just as the SICP/SICG has expanded beyond the oncology context, so too has the operationalization of the SQ in new clinical settings/contexts. Other triggers for serious illness conversations might include prognosis-related triggers (i.e., a ‘no’ response to the SQ), disease/condition-related triggers (i.e., diagnosis of a potentially serious or life-limiting illness), and treatment-related triggers (i.e., initiation or cessation of treatments) [1]. Appropriate and effective patient identification systems have been said to require formation of registries, predictive algorithms, and ongoing clinician education; however, the development, adaptation, and evolution of these new and hybrid identification methods requires evaluation [12,13]. It therefore seems necessary to explore the ways in which patients are currently identified for serious illness conversations to gain a fuller understanding of existent methods, processes, and practices.

The aim of this scoping review is to examine the current literature regarding patient identification for serious illness conversations within the context of the Serious Illness Care Program (SICP) and/or the Serious Illness Conversation Guide (SICG). This review addresses the following research questions:

- How are patients identified for serious illness conversations?
- Who is involved in identifying patients for serious illness conversations?
- How does patient identification for serious illness conversations differ between patient groups and/or clinical contexts?
- What facilitators and/or barriers are described in patient identification for serious illness conversations?

## 2. Materials and Methods

### 2.1. Study Design and Protocol

A scoping review was undertaken to examine the current literature related to patient identification for serious illness conversations within the context of the SICP and/or the SICG. Unlike systematic reviews, scoping reviews support an expansive exploration of a research area to catalogue, map, and synthesize the literature [14,15,16,17,18]. As risk of bias assessments are not typically conducted with this research method, clinical recommendations cannot be made for policy or practice [17,18,19]. Scoping reviews can, however, lead to the identification of knowledge gaps and the formulation of future research questions, and indicate directions for future research studies [14,15,16,17,18]. This scoping review was conducted as per the guidelines set out by The Joanna Briggs Institute [19] and was reported using the Preferred Reporting Items for Systematic Reviews and Meta-Analyses extension for Scoping Reviews (PRISMA-ScR) Checklist [20].

### 2.2. Eligibility Criteria

The eligibility criteria were informed by the study aim and research questions, and were formulated using the Participants, Concept, Context (PCC) Strategy [19]. Using this strategy, the review was limited to the literature that reported identification of human patients (*Participants*) for serious illness conversations (*Concept*) within the *Context* of the SICP and/or the SICG. As the SICP was developed based on a literature review from 2014, only the literature published between (January) 2014 and (September) 2021 was considered for inclusion in this study. This scoping review was not limited by study type or setting; however, the language of publication was limited to English and only peer-reviewed articles were considered (i.e., not conference abstracts or media releases). Sources were excluded if they did not discuss patient identification, or if they discussed serious illness conversations without being related to the Ariadne Labs SICP (i.e., not implementing the SICP and/or not using the SICG or an adaptation).

### 2.3. Search Strategy

A comprehensive search was conducted in September 2021. PsycINFO, Cumulative Index to Nursing and Allied Health (CINAHL), and Medical Literature Analysis and Retrieval System Online (MEDLINE) were interrogated via EBSCOhost, and PubMed was searched separately. Search terms and combinations/truncations were discussed between the authors and developed in consultation with a University Librarian. The search terms were: ‘serious illness program *’, ‘serious illness care’, ‘serious illness conversation *’, ‘serious illness model’, and/or ‘serious illness communication’. Reference lists of included articles were hand-searched. The complete search strategies for each database are detailed in Appendix A.

### 2.4. Selection of Evidence

The initial search results were imported for processing using the bibliographic reference management software EndNote X7.8 for Windows. The first author (RB) conducted the initial title and abstract screening based on the eligibility criteria. The full text was viewed in cases where the title and abstract did not provide sufficient material to inform a decision. Following the initial screening, all articles were read in full and evaluated for inclusion using the same criteria. Another author (AS) reviewed all articles marked for inclusion/exclusion and any uncertainty was discussed between the authors until consensus was reached. Several articles were noted to have originated from overarching study clusters and therefore used the same identification methods; however, these articles were deemed eligible for inclusion as they explored unique study aims, contained different descriptions of patient identification, and illustrated the evolution of how identification methods have changed over time.

### 2.5. Data Charting Process

Data were extracted using charting tables created by the authors, based on the guidelines proposed by The Joanna Briggs Institute [19]. The first charting table collected descriptive information, such as the author(s), publication year, study setting (country, clinical context), study aims, research methods, participants (if applicable), and study results/conclusions. A second charting table was used to collate data regarding patient identification for serious illness conversations, and any additional information relevant to the aim and research questions. Articles were grouped according to their original study cluster and then listed in chronological order of publication year to illuminate the evolution of patient identification methods in SICP/SICG-related research over time. The preliminary data charting tables were piloted on five articles to confirm extraction of relevant information, after which data from the remaining literature were extracted.

### 2.6. Synthesis of Results

A deductive approach was used to organize and summarize information from the literature to address the study aim and questions. Extracted data were compared and contrasted to identify patterns, similarities, and differences in descriptions of patient identification for serious illness conversations. Emerging patterns were organized into categories related to the research questions. These groupings were discussed at length and all authors agreed upon the final results.

## 3. Results

### 3.1. Included Articles

**Figure 1:**
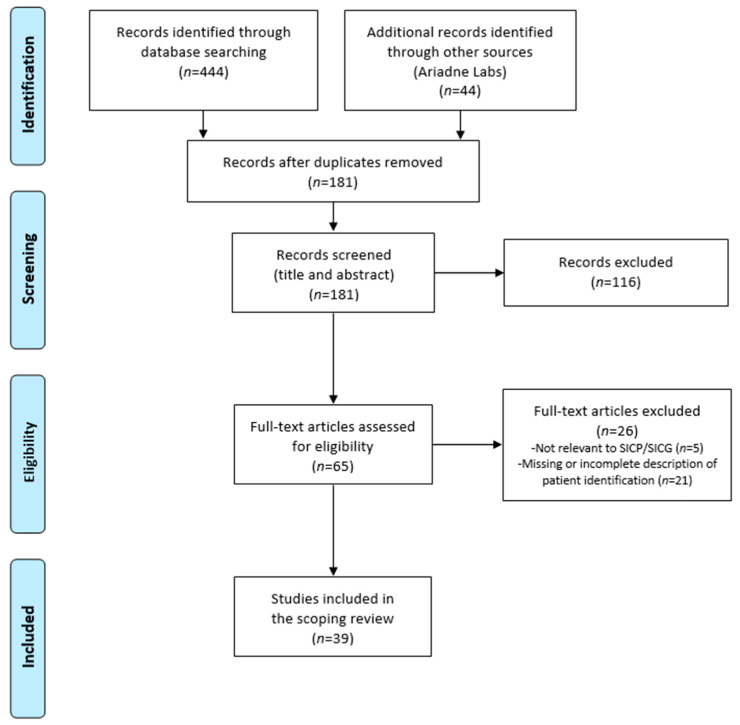
Summary of study selection process—PRISMA-ScR.

The initial database searches returned 444 results (CINAHL *n* = 105; MedLine *n* = 152; PsychInfo *n* = 29; PubMed *n* = 158). A list of 44 articles pertaining to the SICP published by Ariadne Labs was added to the raw list of articles from the initial database search as these articles were directly related to the SICP or SICG. Following the removal of duplicates, 181 articles progressed to title and abstract screening. Of these, 65 met the criteria for full-text review, and 39 met the inclusion criteria for the study. The reference lists of the articles marked for inclusion were examined, and an additional 16 articles were screened at title and abstract level. Of these, three underwent full-text review, but none met the inclusion criteria for the study. In total, 39 articles were eligible for inclusion in this scoping review (Figure 1).

### 3.2. Description of Articles

The articles were set in a range of inpatient and outpatient clinical settings and comprised of staff, patient, and relative participants. A variety of research methods were used, and the majority of studies originated from the United States (*n* = 34). The full characteristics of the included articles are detailed in Appendix B.

### 3.3. Description of Patient Identification

Patient identification for serious illness conversations was described in various ways. Some articles explicitly outlined the entire identification process, clearly stating who was responsible for patient identification, the guidelines for patient identification, the procedures by which patients were identified, the training provided for patient identification (if any), and justification for these procedures. However, in some cases, it was not possible to delineate the separate parts of this process, for example, if it was not specified whether the clinician who held the serious illness conversation was the same person who identified the patient. Detailed descriptions and excerpts regarding how patients were identified and who performed the identification are presented in Table 1.

Almost half of the articles (*n* = 17) described specific clinical- and/or diagnostic-related triggers as their primary method for identifying patients for serious illness conversations. Several articles (*n* = 9) reported using the SQ (one or two years) as their principal identification method, and the remaining articles (*n* = 13) described using some combination of the SQ, clinical/diagnostic-related triggers, patient/family request, and clinician judgement. Physicians were the most frequently named clinicians in the identification process, followed by physician’s assistants, nurse practitioners, medical assistants, nurses, social workers, care coordinators, and allied health staff. Research and administration staff were also said to be actively involved in identifying eligible patients, and several articles indicated that Electronic Health Record (EHR)/Electronic Medical Record (EMR) systems/algorithms were instrumental in the patient identification process.

### 3.4. Patient Identification among Population Groups and Clinical Settings/Contexts

The ways in which patients were identified for serious illness conversations varied across population groups and clinical settings/contexts (Table 2). The SQ (1 or 2 years) was described in the oncology setting (*n* = 7), as were clinical/diagnostic triggers (*n* = 2), and a combination of methods (*n* = 4). Medical (i.e., acute, inpatient, outpatient) and other specialties (i.e., intensive care, pediatrics) clinical settings/contexts primarily identified patients using clinical triggers (*n* = 11). The primary care setting revealed the greatest diversity in identification methods.

### 3.5. Facilitators and Barriers to Patient Identification

Twenty-one articles specified facilitators and/or barriers relevant to patient identification. Potential facilitators were described as including simple and structured screening systems [37], EHR/EMR support and reminders [45], and clinician education [5,29]. Tools such as the SQ were said to improve clinician buy-in and contemplation surrounding recruitment for, and conduction of, serious illness conversations [26]. With regards to barriers, several studies outlined potential discrepancies in the interpretation of identification criteria. Billie and Letizia [39] wrote that there were ‘several situations in which a case manager evaluated the patient as appropriate for an SI [serious illness] conversation, although he or she did not meet the established SI criteria’ (p. 226). Other studies also indicated ambiguity surrounding eligibility criteria, for example, variation in the interpretation of clinical characteristics [34] and differences in understanding what constituted a ‘serious illness’ [50]. Uncertainty surrounding the ideal timing of the conversation, and lack of time to have the conversation, were also stated to be barriers to identification, as recruitment could be limited by patient number or urgency [12,52].

Lakin and colleagues described disparities in the ways in which clinicians identified patients, with staff stating ‘no, we don’t have a process for patient selection’, ‘when I do patient selection, I sit down and look at a list of patients and just choose’, and ‘when I do patient selection, I sit down along with a nurse and we look together at a list of patients choose who needs the conversation’ (p. 760) [27]. It could also be challenging to answer the SQ for patients with multi-morbidities, cognitive impairment, or frailty as life expectancy can vary [29]. Furthermore, among larger, sicker patient groups, the SQ could be inadequate or difficult to operationalize [12]. It was suggested that relying solely on the SQ could overlook some patients who would benefit from a palliative approach [25,26,50]; similarly, replying ‘no’ to the SQ was not always thought to require a serious illness conversation [50]. Triggering criteria for a conversation did not guarantee that a conversation would be held, and without a structured tracking system it could be difficult for clinicians to know who had, or had not, completed serious illness conversations [35,43].

Lack of a systematic approach to identification (i.e., EHR/EMR queries, use of simple trigger thresholds) was said to be a barrier to identifying appropriate patients for serious illness conversations [47]. Studies stated that it could be difficult for clinicians to manually identify patients, particularly when there was no structured EHR/EMR support [12,25,47,48,52]. However, EHR/EMR systems may neglect to flag seriously unwell patients with poor prognoses [31] as not all trigger criteria are available for algorithmic computation [35]. Additionally, it takes time and (human) resources to support such systems [35]. Another potential issue was the efficacy and reliability of EHR/EMR algorithmic triggers, as some have not undergone formal validation and may therefore under- (or over-) identify patients for serious illness conversations [32,33].

## 4. Discussion

This scoping review examined the current literature regarding patient identification for serious illness conversations within the context of the SICP and/or the SICG. The findings revealed that patients were primarily identified using the SQ or clinical/diagnostic triggers. Combinations of criteria and development of automated systems/algorithms indicate ongoing evolution and adaptation of identification methods for different clinical settings/contexts. A diverse range of clinicians was involved in identifying and conducting serious illness conversations, with physicians, nurses, and automated EHR/EMR systems the most commonly named actors in the identification process. Barriers and facilitators were described regarding clinician understanding of the concepts and identification criteria, structured support systems, and training/education.

In recent years, the SQ has emerged as a useful screening tool to identify patients nearing the end of life who may benefit from a palliative approach to care. A major advantage of the SQ is that it encourages a level of closeness between the clinician and the patient, prompting active contemplation of the patient’s unique situation and care needs [26]. It is, however, important to note that the SQ has reported mixed sensitivity (low to reasonable/good), and responses to the question are said to be impacted by the clinician’s familiarity with both the question and the patient [50,55,56,57]. Furthermore, repeatedly asking oneself the SQ is not only time consuming but can be emotionally exhausting given the gravity of the overarching topic [12,58]. These findings revealed how the use of the SQ in the SICP/SICG has evolved over time with 1- and 2-year alternatives, and combinations with clinical/diagnostic triggers, clinician judgement, and patient/family factors. Nevertheless, the efficacy of these adaptations and combinations to accurately identify patients for serious illness conversations has not yet been established.

The results show that clinical/diagnostic triggers have emerged as a popular identification method, particularly in acute and specialty clinical contexts. These criteria ranged in specificity from targeted lab values, to entire patient populations. As various phases of illness are often distinguished by changes in function, pain, perception, or physical ability, monitoring of clinical/diagnostic triggers provides valuable information to inform patient identification at the so-called ‘right’ time [59,60]. This is important because if (mis)identification occurs too early or too late in the illness trajectory it can result in undue physical, mental, emotional, and spiritual labor for both patients and clinicians [61]. However, according to Kelley and Bollens-Lund [2], identifying seriously ill patients using administrative data alone (i.e., diagnosis codes, hospitalizations) is not sufficient. This may support the use of combined methods for identification, such as prognosis-related triggers and indicators of critical loss, or clinical/diagnostic triggers and calculations of resource use [13,55,62]. Further research would therefore be useful to compare and contrast clinical/diagnostic triggers between specialties and explore the effectiveness of different combinations and hybrid methods in identifying patients for serious illness conversations.

This study found that of the numerous clinicians named in the identification process, physicians were the most common identifiers of patients for serious illness conversations. Other clinicians or non-clinical resources that were described in this process included physician’s assistants, nurse practitioners, medical assistants, nurses, social workers, care coordinators, allied health staff, researchers, and EHR/EMR applied algorithms. It appears that the roles and responsibilities in relation to patient identification have evolved over time to include a more diverse range of clinicians and resources. However, in some articles it was not stated who performed the identification, and in others it was unclear if the clinicians who received SICP training performed the patient identification and held the subsequent conversation. Transparency was lacking regarding *when* in the care trajectory patients were considered for serious illness conversations, nor were there extensive justifications as to *why* particular triggers were selected or excluded. For example, it is interesting to note that few studies included patient/family benefit or readiness as a criterion for these conversations. Perhaps this is because it is still unclear whether the ‘tipping point’ for recognition of seriously ill patients is more closely linked to demographics, diagnosis, symptoms, prognosis, clinical context, and critical loss, or to the clinicians’ own perceptions and experiences [62]. This reinforces the need to develop identification protocols that provide specific guidance regarding health/illness trajectories and their associated conversations [2,63]. It also seems important to distinguish (and report) each step in the identification process, namely (1) how potential patients are identified; (2) how this information is communicated to clinicians; and (3) how clinicians evaluate patient eligibility (and readiness) for conversations. While this scoping review only explored part of this process, it would be useful for future studies to examine how clinicians justify decisions regarding patient eligibility for serious illness conversations, including motivations as to why they did or did not initiate a conversation in practice.

### Limitations

This scoping review has several limitations. First, the review was limited to the literature that described patient identification for serious illness conversations in the context of Ariadne Labs’ SICP/SICG. Studies that used the SICP and/or the SICG, but did not describe patient identification, or that described patient identification but did not explicitly state their affiliation with the SICP/SICG, were therefore excluded or omitted. Other studies pertaining to serious illness conversations that used different conversation programs, tools, or guides may outline different identification methods. As the SICP informs the SICG, and vice versa, we did not separate the included articles into groups that used the SICP only, the SICG only, or some form of adaptation. Further, while the majority of studies in this review originated from the United States, a recent survey of the Serious Illness Care Community of Practice indicated that the program and the guide have been implemented in a wide range of clinical settings across 45 countries [11]. Research and publications from outside North America regarding the SICP and SICG are therefore ongoing.

It should also be noted that the number of articles written about the SICP/SICG outnumber the number of unique studies. This is because the SICP and SICG originated from a research group based out of Ariadne Labs, a joint center for health systems innovation at Brigham & Women’s Hospital and the Harvard T.H. Chan School of Public Health, and has been subsequently adopted for implementation at several other clinical/research sites (i.e., Massachusetts General Hospital, the University of Pennsylvania Health System, etc.). These articles were reported and analyzed individually due to differences in descriptions of identification methods and changes to identification methods that may have occurred over time. The relationship between the articles and study clusters is highlighted in the tables and footnotes for transparency.

Finally, a distinguishing feature of scoping reviews is their focus on providing a broad overview of the existing literature, irrespective of type or quality; hence, a formal evaluation of the risk of bias of the included articles was not undertaken [17,18,19]. As such, these findings are exploratory/descriptive in nature and do not seek to explain or analyze the literature in relation to policy or practice [17]. This study did, however, take care to provide a detailed description of the characteristics of the included literature so it is left to the reader to decide the generalizability and relevance of these findings.

## 5. Conclusions

The findings from this scoping review shed light on current methods, processes, and practices used to identify patients for serious illness conversations in the context of the SICP and/or the SICG. Identification methods varied among different clinical settings/contexts and included the SQ, clinical/diagnostic triggers, and combinations of criteria. A constellation of clinicians and resources were described in the identification process. Although this study provides an initial understanding of the existent patient identification methods for serious illness conversations, reporting methods for identification were inconsistent and there appears to be a lack of validated and standardized protocols for comparison. As timely patient identification is arguably one of the most challenging components of the SICP/SICG, future research is necessary to explore how clinicians justify and motivate decisions regarding patient identification and to establish the efficacy of these adapted/combined identification methods.

## Figures and Tables

**Table 1:** Description of patient identification ^†^.

Article	Method	How Patients Were Identified for Serious Illness Conversations (Actual or Planned)	Who Identified the Patient (Actual or Planned)
Dana-Farber Neuro-oncology Pilot Cluster (*n* = 2)			
Bernacki et al. (2015) [6]	SQ (1 year) and clinical/diagnostic triggers	To identify eligible patients, we use a ‘No’ answer to the SQ (p. 5). Recruitment in the neuro-oncology clinic also included a review of ICD-9 codes to identify patients with a diagnosis of a cancer that has a high mortality risk (e.g., glioblastoma multiforme) (p. 6).	Patients are identified by a **clinician** [physicians, physician assistants, nurse practitioners] (p. 5). Trained **clinicians** were triggered by the **research staff** to have the SICG discussion with enrolled patients (p. 7).
Miranda et al. (2018) [21]	SQ (1 year) and clinical/diagnostic triggers	Patients were screened for inclusion either by chart review for a documented diagnosis of glioblastoma, OR by asking their clinician the SQ. Patients with a documented glioblastoma diagnosis, or for whom the answer to the SQ was ‘no’, were eligible (p. 805).	Not explicitly stated who conducted chart review. **Clinician** [physicians, nurse practitioners] answered the SQ (p. 805).
**Dana-Farber Cluster Randomized Trial Cluster (*n* = 6)**			
Geerse et al. (2019) [4]	SQ (1 year)	Clinicians systematically used the SQ to identify eligible patients with advanced cancer whom they believed were at risk of dying within one year (p. 774).	**Clinicians** [physicians, physician assistants, nurse practitioners] (p. 776).
Paladino et al. (2019) [5]	SQ (1 year)	The SQ was applied at regular intervals by oncology clinicians to lists of their patients (p. 803).	**Oncology clinicians** [physicians, nurse practitioners, and physician assistants] (p. 803).
Bernacki et al. (2019) [3]	SQ (1 year)	Enrolled oncology clinicians identified eligible patients by reviewing patient lists at regular intervals and answering the SQ. Patients for whom clinicians responded no were eligible for participation (p. 752).	Enrolled **oncology clinicians** [physicians, physician assistants, nurse practitioners] (p. 752).
Paladino et al. (2020) [22]	SQ (1 year)	Eligible patients were… identified by their oncology clinician with a ‘no’ response to the SQ (p. 4551).	**Oncology clinicians** [advanced practice clinicians and physicians] (p. 4551).
Paladino et al. (2020) [23]	SQ (1 year)	To identify eligible patients… all clinicians used SQ. Only patients for whom the clinician responded ‘no, I would not be surprised’ were eligible (p. 1366).	All **clinicians** [advanced practice clinicians and physicians] used the SQ (p. 1366).
Sanders et al. (2020) [24]	SQ	Systematic identification of patients using the SQ (p. 890).	Not explicitly stated who identified patients. **Physicians and advance practice providers** to have conversation (p. 890).
**Brigham Primary Care Integrated Care Management Program Cluster (*n* = 5)**			
Lakin et al. (2017) [25]	SQ (2 years) and clinician judgement	Clinicians each answered the SQ… Clinicians could also add patients to and remove them from their lists based on their clinical judgment (p. 1260).	Patients were identified by **clinicians** [physicians, nurse care coordinators, social workers]. The **implementation team** distributed lists of identified patients every other week to the **nurse care coordinator** who helped coordinate conversation timing (p. 1260).
Lakin et al. (2019) [26]	SQ (2 years) and clinical/diagnostic triggers	Primary care providers reviewed lists of eligible patients and select the most appropriate patients to enroll in the iCMP. Then, to identify which iCMP patients were eligible for the SICP, primary care physicians and nurse care coordinators answered the 2-year SQ (p. 1468).	**Primary care physicians** and **nurse care coordinators** identified patients for serious illness conversations (p. 1468).
Lakin et al. (2019) [27]	Clinician judgement	Patient screening: The interviewee is discussing their process for how they do patient selection or identification—‘When I do patient selection, I sit down and look at a list of patients and just choose.’ Spontaneous patient selection: The interviewee talks about times when they have a conversation with a patient organically, rather than planned in advance—‘Sometimes I am talking to the patient and I realize that it’s just time to have the conversation’ (p. 760).	**Primary care clinicians** [physicians, nurses, social workers] (p. 752).
Lakin et al. (2020) [28]	SQ (2 years) and clinical/diagnostic triggers	Patients who had been enrolled in the iCMP, had complex medical histories, and were well-known to the clinicians who identified them for a serious illness conversation via electronic surveys using the SQ (p. 100431).	**Clinicians** [physicians, nurses, ‘other clinicians’—unspecified] identified patients for conversations (p. 100431).
Paladino et al. (2021) [29]	SQ (1 year), clinical/diagnostic triggers, and patient/family request	Clinicians described three approaches to selecting patients for conversations: (1) Use of the SQ by reviewing lists of patients identified as high-risk (2) Response to a triggering medical event or assessment of the patient’s health status, which led clinicians to initiate a discussion; (3) Responding to patient- or family-initiated statements that clinicians interpreted as a sign of readiness for the conversation (p. 461).	**Clinicians** [primary care physicians, nurse care coordinators, and social workers] (p. 460).
**University of Pennsylvania Machine Learning Cluster Randomized Trial (*n* = 2)**			
Manz et al. (2020) [30]	Clinical/diagnostic triggers	An EHR-based machine learning algorithm uses real-time patient data, including demographic information, comorbidities, lab values, and encounters with the health system over the prior six months, to estimate individuals’ risk of dying in the subsequent six months (p. 2). Clinicians could view a list of up to six patients scheduled for a visit in the coming week with the highest-risk of machine-predicted six-month mortality (p. 4).	**EHR-based machine learning algorithm** estimated individuals’ risk of dying in the subsequent six months. Then patient selection by **clinicians** [medical oncologists, nurse practitioners, physician assistant] (p. 2).
Manz et al. (2020) [31]	Clinical/diagnostic triggers	Clinicians could review a list of patients scheduled for the following week in their clinic who had a high risk of mortality. Mortality risk was determined by a machine learning algorithm, which used structured EHR data to predict risk of 180-day mortality. Clinicians could view a list of up to 6 patients with the highest predicted 180-day mortality risk (p. 3).	**Clinicians** [physicians, nurse practitioners, physician assistants] could review patients scheduled for the following week in their clinic who had a ‘high risk’ of mortality (p. 2).
**Massachusetts General Hospital Cluster (*n* = 2)**			
Gace et al. (2020) [32]	Clinical/diagnostic triggers	An automated, EHR embedded screening tool identified patients at increased risk for unmet palliative needs. This Epic algorithm scanned the patient registry, problem list and progress notes to identify inpatients with high-risk diagnoses; limited prognosis; and language regarding the need for advance care planning, palliative care, or family meetings. Patients who met any criteria were considered to have a positive screen and were said to have ‘triggered’ the tool (p. 1494).	An **EHR-embedded screening tool** identified patients at increased risk for unmet palliative needs. The **research assistant** would review all new admissions to determine patients who screened positive. The **research assistant** notified the **clinicians** [doctor, nurse practitioner, physician assistant, nurse, case manager, social worker] about these patients and asked **clinicians** to consider whether a serious illness conversation would be appropriate (p. 1494).
Greenwald et al. (2020) [33]	Clinical/diagnostic triggers	An automated electronic screening tool identified patients who were at risk for potential unmet palliative care needs (p. 1501). Hospitalists on intervention units received verbal notification when their recently admitted patients were identified using a computer algorithm as having possible unmet palliative needs. Hospitalists on the control unit received no notifications (p. 1500).	A **research assistant** reviewed recent admissions to identify patients who triggered the **automated screening tool**. The research assistant would inform **clinicians** [physicians, physician assistants, nurse practitioners] that the patient had been identified as possibly having unmet palliative needs and recommend that the clinician consider initiating a serious illness conversation (p. 1501).
**Stand-alone studies not part of clusters (*n* = 22)**			
Lamas et al. (2017) [34]	Clinical/diagnostic triggers	We defined chronically critically ill patients as those who had undergone tracheotomy for prolonged mechanical ventilation. The admitted critical chronic illness patients were screened, and patients or surrogates were approached within two weeks of admission (p. 712).	**Researcher** recruitment of patients, with permission from the attending **clinician** (p. 712).
Massman et al. (2019) [35]	Clinical/diagnostic triggers	Primary triggers (1 or more): Any metastatic solid tumor; COPD with home O2 and/or FEV1 < 35% predicted; History of CHF; CKD Stage IV or V; Chronic liver disease with cirrhosis and/or ascites; Age > 90 years. Secondary triggers: A1c > 8.5; >2 emergency department visits and/or hospitalizations in past 6 months; Functional decline; Cognitive status; Noncompliance; Age > 80 years (p. 293).	A report function was built in the **EHR system** to generate a list of patients who were scheduled for an appointment with a **primary care provider** at the clinic site and met primary trigger criteria. This report, run weekly by a **clinic Registered Nurse**, identified the patients meeting primary and secondary triggers (p. 292).
Mandel et al. (2017) [36]	SQ (1 year), clinical/diagnostic triggers, and person/family request	Before dialysis—Not surprised in answer to SQ; High likelihood of progression to ESRD; Dialysis modality teaching referral; Access referral; Access placement; Transplant referral; Recurrent or prolonged hospitalizations; Changes in function or dependence; Sentinel events or indicators; Patient or family request (p. 856). After beginning dialysis—Not surprised in answer to SQ; Access procedures; Recurrent or prolonged hospitalizations; Changes in function or dependence; Sentinel events or indicators. Admission to the dialysis unit; After three months on dialysis; Annually; Patient or family request (p. 856).	Not explicitly stated who would undertake identification. Article outlines that patients generally expect such conversations to be initiated by their **clinician** (suggested: nephrologists, dialysis nurses, social workers, and primary care physicians) (p. 855).
O’Donnell et al. (2018) [37]	SQ (1 year) and clinical/diagnostic triggers	Patients currently or recently hospitalized with at least one poor prognostic indicator (p. 517): hospitalization for heart failure management within a year prior to the index hospitalization; age ≥ 80 years; advanced CKD; SBP ≤ 100 mm Hg; serum sodium ≤ 130 mEq/L; cardiogenic shock; serious non-cardiovascular illness limiting 1-year life expectancy: using SQ (Supplement 2, p. 2).	**Physician** expectations of prognosis were queried using the SQ. All patients were identified and enrolled by the **study coordinator** (p. 517).
Totten et al. (2019) [38]	SQ (2 years) and clinical/diagnostic triggers	Patients may have any serious illnesses or conditions that are likely to limit their life expectancy to less than two years as defined by using clinical intuition (e.g., SQ) alone, or supplemented by an available algorithm (mortality index) (p. S-85).	Clinician-focused model: the **primary care clinician** [physicians, nurse practitioners, or physician assistants] identifies appropriate patients. Team-based model: **primary care team members** share SICP tasks appropriate to their scope of practice [a primary care clinician and, for example, nurses, care managers, social workers, medical assistants, chaplains, peer counselors, community health workers, etc.] (p. S-83).
Billie and Letizia (2020) [39]	Clinical/diagnostic triggers	Unplanned inpatient admission in the last six months; And one or more of the following: Cancer (poor prognosis, metastatic or hematological); COPD or interstitial lung disease (only if using home oxygen or hospitalized); Renal failure (end stage); Congestive heart failure (only if hospitalized); Advanced liver disease or cirrhosis; Diabetes with severe complications (p. 225).	Transitions- of-care referrals were identified daily from an **EMR report** and assigned to the respective **case managers** [nurses and social workers]. **Case managers** identified patients as being appropri ate for a serious illness conversation. **The Project Director** validated that the patient met criteria (p. 225).
Kumar et al. (2020) [40]	SQ (1 year)	Patients were considered eligible if clinicians answered ‘no’ to the SQ (p. e1508).	**Oncology clinicians** [physicians, nurse practitioners, physician assistants] were encouraged to choose 1 to 2 patients per week with whom to have a serious illness conversation (p. e1508).
Lakin et al. (2020) [12]	SQ (1 and 2 year) and clinical/diagnostic triggers	University of Pennsylvania Health System: Printed weekly patient schedules for physician review using the SQ with a 1-year duration. Later changed to system where provider selected patients ad hoc (p. 2). Baylor Scott & White Health: List created using ICD-10 codes for specific illnesses, multiple comorbidities, multiple hospitalizations in the prior year and payor type. Followed by clinician review using the SQ of varying duration (p. 2). Stanford Healthcare: Manual chart review to identify patients with specific clinical characteristics (p. 4). National Health Service, U.K.: The program team coded lists of patients as receiving palliative treatment. Clinicians reviewed their individual lists of pre-screened patients and used the SQ to identify those felt to be at risk of death in the next 1–2 years. At primary care sites, general practitioners used a practice register of patients thought to be in the last 12 months of life to identify patients who they felt should be offered a serious illness conversation (p. 5). Brigham Health: Initially, screened patients deemed eligible for the iCMP were asked the SQ on a paper survey as part of the enrolment process, but this missed many patients. The second patient selection algorithm expanded the timeframe of the SQ to 2 years and asked it as part of a SICP-specific electronic screening survey (p. 6).	University of Pennsylvania Health System: (1) **physician, advance practice provider, medical assistant** review of patient schedule, (2) free **provider** choice (p. 3). Baylor Scott & White Health: (1) ICD-10 codes on the **EHR**. SICP **program managers** and **local practice administrators** approached **physicians** with the SQ; (2) **hospitalists and case managers** used the SQ on all admitted patients; (3) **oncology nurses** collaborated with **physicians** to identify patients (p. 4). Stanford Healthcare: Patients were identified by a **nurse coordinator** and **research assistant** (p. 5). National Health Service, U.K.: (1) **General practitioners** identified patients; (2) **Clinicians** reviewed patient lists using the SQ; (3) **Interprofessional teams**, including **clinical nurse specialists**, **allied healthcare professionals** and **administrative staff**, played important roles in patient selection and workflow organization (p. 5). Brigham Health: (1) In the pilot selection process a **clinician** screen asked **primary care doctors** the SQ; (2) The **patient selection algorithm** expanded the SQ and asked it as part of an **electronic screening survey** sent to **doctors, care coordination nurses and social workers** (p. 6).
Lally et al. (2020) [41]	Clinical/diagnostic triggers	A daily dashboard identifies when ACO patients are admitted to the hospital, and patients who meet the criteria for CCM were enrolled. Any patient identified on this daily report is added to a spreadsheet and the data analyst looks for a documented serious illness conversation within 14 days of discharge from the hospital (p. 113).	A **dashboard** identified when ACO patients are admitted. Then **nurse case managers** enrolled patients who met the criteria (p. 113).
Ma et al. (2020) [42]	Clinical/diagnostic triggers	Patients were eligible to be enrolled in the SICP if they were admitted to a medical ward, had a stay of at least 48 hours, and received a score of 5 or 6 on the interRAI Emergency Department Screener on admission (p. E449).	The **unit champion** (former bedside **nurse** from the medical ward) screened medical inpatients for eligibility. The **unit champion** triggered **clinicians** to have the conversations (p. E449).
Pasricha et al. (2020) [43]	Clinical/diagnostic triggers	Providers met with surrogates of adult, mechanically ventilated patients in the medical intensive care unit within 48 hours of intubation (p. 120).	Not explicitly stated who identified patients. **Providers** [physicians and hospitalists] to have conversation (p. 120).
Pottash et al. (2020) [44]	Clinical/diagnostic triggers	Patients with a chronic, serious illness were identified by hospital record search using the following criteria: (1) admitted in the previous six months for either lung disease, liver disease, heart failure, or stroke/dementia; and, (2) a physician trainee had written a note in their chart (p. 1188).	Patients were identified by **hospital record search** (not stated who performed search). Second- and third-year **internal medicine trainees** to have conversation (p. 1188).
Wasp et al. (2020) [45]	Clinical/diagnostic triggers and clinician judgement	Fellows identified a range of patients who they felt were appropriate candidates for a serious illness conversation: patients within hours to days of death, to those with incurable cancer failing treatment, and those with personal or family emotional distress (p. 4).	**Fellows** identified patients (p. 4).
DeCourcey et al. (2021) [46]	Clinical/diagnostic triggers	The preliminary PediSICP intervention [was] tentatively triggered by prolonged inpatient hospitalization (>2 weeks) or a hospital readmission (p. 248).	Patient and parent participants were either **self-referred**, or referred by the **palliative care service**. Participants not self-referred were approached in person, after gaining **attending approval**, and invited to participate (p. 248).
Hafid et al. (2021) [47]	Clinical/diagnostic triggers	Patients aged 65 or older with any diagnosis of a chronic, progressive illness or frailty that is expected to decrease life expectancy (p. 3).	**Primary care providers** [physicians, nurse practitioners, registered nurses, social workers] (p. 2).
Karim et al. (2021) [48]	SQ (1 year) and clinical/diagnostic triggers	The patient met one or more of the following criteria: a response of ‘no’ to the SQ, any patient with a diagnosis of metastatic pancreatic cancer, or symptom scores of >7 on more than three categories on our patient-reported outcome dashboard (p. 906).	The **clinician** [medical oncology physicians] together with their **primary nurse** was asked to identify at least one patient that week who would be appropriate for a serious illness conversation (p. 906).
Lakin et al. (2021) [49]	Clinical/diagnostic triggers	Patients were screened using pre-defined EMR-based criteria, which included attribution to the Brigham & Women’s Hospital ACO, in addition to one of two additional clinical criteria: (1) age over 80, or (2) age 75–79 with two or more admissions in the preceding six months (p. 2).	Screened by pre-defined **EMR-based criteria**. The **social worker** supported **clinicians** [physicians, nurses, physician assistants] to identify and assess patients’ readiness for a conversation (p. 2).
Le et al. (2021) [50]	SQ (1 year)	The original criteria to indicate a serious illness conversation was that only one team member had to not be surprised if a patient died within the next year. Feedback from some staff indicated they would prefer to be in full agreement to indicate a conversation. Thereafter, all team members needed to be in agreement about the SQ answer (p. 1014).	Patients were identified during **interdisciplinary team** care rounds with doctors, clinical teaching unit staff, nurses, and allied health staff.
Paladino et al. (2021) [51]	Clinical/diagnostic triggers	Outpatient setting: clinicians to proactively reach out to patients in the community with underlying health conditions who are at higher risk of serious complications should they contract COVID-19. Inpatient setting: clinicians to have ACP conversations with patients admitted to the hospital with confirmed or suspected COVID-19 (or their families) (p. 129).	Outpatient setting: **clinicians** to proactively reach out to patients who are at higher risk of serious complications should they contract COVID-19. Inpatient setting: **clinicians** to have conversations with patients admitted to the hospital with confirmed or suspected COVID-19 (p. 129).
Schmidt et al. (2021) [52]	Clinical/diagnostic triggers	Eligible patients must: be seriously ill or frail; be expected to live 1 to 2 years; and, have participated in an ACP conversation with trained clinicians and nursing staff. Marking patients on the office schedule for clinicians using the Gagne Index (p. 2). Trigger: Mortality score of 14.6% or higher (p. 3).	**Clinicians, physicians, and nursing staff**. Three participating offices used the **EMR**. Staff indicated it would be helpful if **the researchers** could identify eligible patients who were scheduled for upcoming office visits (p. 2).
Swiderski et al. (2021) [53]	SQ (2 years)	Physicians identified patients using a modified SQ (p. 2).	**Physicians** [family medicine attending physicians] identified patients (p. 2).
Thamcharoen et al. (2021) [54]	Clinical/diagnostic triggers	Patients with CKD stage ≥ 3B with the following criteria: age ≥ 80 years or; age ≥ 70 years with diabetes or cardiovascular disease or; any age with other advanced stage organ diseases, such as: heart failure with New York Heart Association class III or IV, severe COPD, cirrhosis with child class C or Model for End-Stage Liver Disease score ≥ 17, any age with metastatic cancer, or any age with CKD stage 4 or 5 (p. 3).	Not explicitly stated who identified patients. Participants completed the adapted SICG in person with a **study investigator** who had completed a SICG training course (p. 2).

^†^ All data originated from, or was adapted from, the associated source indicated in the table. Abbreviations: A1c—glycated hemoglobin; ACO—Accountable Care Organization; ACP—Advance Care Planning; CCM—Complex Care Management; CHF—Chronic Heart Failure; CKD—Chronic Kidney Disease; COPD—Chronic Obstructive Pulmonary Disease; EHR—Electronic Health Record; EMR—Electronic Medical Record; ESRD—End-Stage Renal Disease; FEV1—Forced Expiratory Volume 1 second; ICD—International Classification of Diseases; iCMP—Integrated Care Management Program; interRAI—International Resident Assessment Instrument; mmHG—millimeters of mercury; mEq/L -milliequivalents per liter; O2—Oxygen; SBP—Systolic Blood Pressure; SQ—Surprise Question; U.K.—United Kingdom.

**Table 2:** Identification methods among population groups and clinical settings/contexts *.

Clinical Setting/Context ^†^	SQ	Clinical/Diagnostic Triggers	Clinician Judgement	SQ and Clinical/Diagnostic Triggers	SQ and Clinician Judgement	SQ and Clinical/Diagnostic Triggers and Patient/Family Judgement	Clinical/Diagnostic Triggers and Clinician Judgement
Oncology-Inpatient/Outpatient	(3, 4, 5, 22, 23, 24) ^‡^, 40	(30, 31) ^¶^		(6, 21) ^§^, 48			45
Primary Care-Urban/Rural		35, 39, 52	(27) ^‖^	(26, 28) ^‖^, 38	(25) ^‖^	(29) ^‖^	
Medical-Inpatient/Outpatient	50	(32, 33) ^††^, 34, 37, 42, 47, 49					
Intensive Care		43					
COVID-19		51					
End-stage renal failure/Nephrology		54				36	
Pediatrics		46					
Community Care/Health	53	41					
Ambulatory Care		44					

* Sources in brackets denote connection to a study cluster, indicated in the table footnotes; ^†^ Lakin et al. [12] not listed due to multiple clinical settings and identification methods; ^‡^ Dana-Farber Neuro-oncology Pilot Cluster; ^§^ Dana-Farber Cluster Randomized Trial Cluster; ^‖^ Brigham Primary Care Integrated Care Management Program Cluster; ^¶^ University of Pennsylvania Machine Learning Cluster Randomized Trial; ^††^ Massachusetts General Hospital Cluster.

## Data Availability

The full dataset of included studies is available.

## Appendix A

**Table A1:** Database search strategy.

Database	Search Strategy
CINAHL	TI (“serious illness communication” OR “serious illness program *” OR “serious illness care” OR “serious illness conversation *” OR “serious illness model”) OR AB (“serious illness communication” OR“serious illness program *” OR “serious illness care” OR “serious illness conversation *” OR “serious illness model”) Limiters: Date of publication 20140101-20210901; English language
MedLine	TI (“serious illness communication” OR “serious illness program *” OR “serious illness care” OR “serious illness conversation *” OR “serious illness model”) OR AB (“serious illness communication” OR“serious illness program *” OR “serious illness care” OR “serious illness conversation *” OR “serious illness model”) Limiters: Date of publication 20140101-20210901; English language
PsychInfo	TI (“serious illness communication” OR “serious illness program *” OR “serious illness care” OR “serious illness conversation *” OR “serious illness model”) OR AB (“serious illness communication” OR “serious illness program *” OR “serious illness care” OR “serious illness conversation *” OR “serious illness model”) OR KW (“serious illness communication” OR “serious illness program *” OR “serious illness care” OR “serious illness conversation *” OR “serious illness model”) Limiters: Date of publication 20140101-20210901; English language
PubMed	((((“serious illness communication”[Title/Abstract]) OR (“serious illness program *”[Title/Abstract])) OR (“serious illness care”[Title/Abstract])) OR (“serious illness conversation *”[Title/Abstract])) OR (“serious illness model”[Title/Abstract]) Limiters: Date of publication 20140101-20210901; English language

Note: The terms “serious illness program *” and “serious illness model” were not recognized by PubMed.

## Appendix B

**Table A2:** Characteristics of the included literature ^†^.

Author/s, Year, Country	Aim/s	Design/Methods/Context	Participant Characteristics		Results/Conclusions
			Participants N (Age) *	Female N (%)	
Bernacki et al. (2015) ^‡^ [6] U.S.	This article describes the protocol for a cluster randomized controlled trial of a multicomponent, structured communication intervention.	Study protocol, prospective, cluster randomized controlled trial. Oncology.	-	-	We believe that developing scalable models for improving SICs will contribute to better alignment of healthcare with the preferences of oncology patients, and eventual extension to other patient populations and care settings.
Lakin et al. (2017) ^¶^ [25] U.S.	Describes the implementation of the program and our evaluation of the use of the program by clinicians and the intervention’s impact on the prevalence, timing, accessibility, and comprehensiveness of documented SICs and hospice use among patients.	Prospective implementation trial. Primary care.	Patients: Int. = 101 (79.5 y) Comp. = 77 (78.5 y)	46 (45.6%) 42 (54.5%)	Patients in the clinics with the program implemented were more likely than those in comparison clinics to have SICs-including discussion of values and goals-documented in patients’ medical records. Clinicians who participated also reported high satisfaction with training they received as part of the program, which they regarded as effective.
Lamas et al. (2017) [34] U.S.	To determine the feasibility, acceptability, and potential usefulness of conversations about serious illness with chronic critical illness patients or their surrogate decision makers after LTACH admission.	Exploratory pilot study. LTACH.	Patient = 30 Surrogate = 20	10 (33%) 10 (50%)	Conversations about serious illness care goals can be accomplished in a relatively short period of time, are acceptable to chronically critically ill patients and their surrogate decision makers in the LTACH, and are perceived as worthwhile by patients, surrogates, and clinicians.
Mandel et al. (2017) [36] U.S.	(To) (1) identify the barriers to SICs in the dialysis population, (2) review best practices in and specific approaches to conducting SICs, and (3) offer solutions to overcome barriers as well as practical advice, including specific language and tools, to implement SICs in the dialysis population.	Special issue article. End-stage renal disease.	-	-	Implementing SICs for patients on dialysis involves identifying patients at the highest risk of adverse outcomes, triggering conversations, and conducting them routinely. The Guide provides a tested, scalable structure for conducting these conversations that can be used by nephrologists and other dialysis clinicians, and it can be adapted further to meet the needs of this population. Documentation and sharing of conversation content and identification of metrics to drive performance improvement are also essential to the successful implementation of SICs for patients on dialysis.
Miranda et al. (2018) ^‡^ [21] U.S.	To describe the prevalence, timing, and quality of documented SICs and evaluate their focus on patient goals/priorities.	Retrospective chart review, descriptive. Oncology.	Staff = 6 Patients = 33	3 (50%) 14 (42%)	Patients with GBM had multiple goals/priorities with potential treatment implications, but documentation showed SICs occurred relatively late and infrequently reflected patient goals/priorities.
O’Donnell et al. (2018) [37] U.S.	To determine if early initiation of goals of care conversations by a palliative care-trained social worker would improve prognostic understanding, elicit advanced care preferences, and influence care plans for high-risk patients discharged after HF hospitalization.	Prospective randomized clinical trial. Cardiology.	Patients = 50 Int. = 26 (74.7 y) Cont. = 24 (69.2 y)	12 (46.2%) 9 (37.5%)	Without an adverse impact on quality of life, prognostic understanding, and patient–physician communication regarding goals of care may be enhanced by a focused, social worker–led palliative care intervention that begins in the hospital and continues in the outpatient setting.
Geerse et al. (2019) ^‖^ [4] U.S.	To characterize the content of SICs and identify opportunities for improvement.	Cluster randomized trial, descriptive qualitative. Outpatient oncology.	Staff = 16 Patients = 25 (60.4 y)	8 (50%) 12 (48%)	Exploratory data from this subset of the Dana-Farber cluster-randomized trial suggest that seriously ill patients are open to discussing values and goals with their clinician. Yet, clinicians may struggle when disclosing a time-based prognosis and in responding to patients’ emotions.
Lakin et al. (2019) ^¶^ [27] U.S.	To explore the perceptions of primary care clinicians about interprofessional work in serious illness communication.	Descriptive qualitative. Primary care.	Staff = 14	10 (71.4%)	This study suggests three key areas of focus for design and implementation of programs aimed at improving SICs by interprofessional primary care teams: establishing clear professional roles and responsibilities, paying special attention to interprofessional and clinician-patient relationships, and clearly structuring interventions aiming to change the way our system drives serious illness communication.
Paladino et al. (2019) ^‖^ [5] U.S.	To evaluate the efficacy of a communication quality-improvement intervention in improving the occurrence, timing, quality, and accessibility of documented SICs between oncology clinicians and patients with advanced cancer.	Cluster randomized clinical trial. Outpatient oncology.	Staff Int. = 37 Cont. = 39 Patients Int. = 76 (62 y) Cont. = 85 (63 y)	23 (62%) 20 (51%) 41 (54%) 47 (55%)	This communication quality-improvement intervention resulted in more, earlier, better, and more accessible SICs documented in the EMR.
Bernacki et al. (2019) ^‖^ [3] U.S.	To examine feasibility, acceptability, and effect of a communication quality-improvement intervention (SICP) on patient outcomes.	Cluster randomized clinical trial. Outpatient oncology.	Staff = 91 Patients = 278	52 (57.1%) 148(53.2%)	The results of this cluster randomized clinical trial were null with respect to the co-primary outcomes of GCC and peacefulness at the end of life. However, the significant reductions in anxiety and depression in the intervention group are clinically meaningful and require further study.
Lakin et al. (2019) ^¶^ [26] U.S.	To evaluate the effectiveness of a clinician screening tool to identify patients for a communication intervention.	Prospective cohort study. Primary care.	Staff: Physician = 66 Nurse = 16 Patients: PSQ = 1163 (70.1 y) NSQ = 1148 (69.8 y)	37 (56.1%) 15 (93.8%) 663(57.4%) 871(60.4%)	When used in combination with a high-risk algorithm, the 2-year version of the SQ captured the majority of patients who died, demonstrating better than expected performance as a screening tool for a serious illness communication intervention in a heterogeneous primary care population.
Totten et al. (2019) [38] U.S. and Canada	We are conducting a cluster randomized trial comparing team-based to clinician-focused ACP using the SICP.	Protocol for a cluster randomized trial. Primary care.	-	-	Our dissemination will report the results of comparing the two models and the implementation experience of the practices to create guidance for the spread of ACP in primary care.
Massman et al. (2019) [35] U.S.	To provide a structure within a primary care clinic to facilitate conversations with seriously ill individuals about their care preferences.	Implementation study. Rural primary care outpatient clinic.	Staff = 5 Patients = 22	4 (80%) -	Provider perceptions of conversations after implementation were positive. During the pilot, 3 SICs were initiated with additional patients prepared for future conversations using an information sheet and introduction to the conversation.
Manz, et al. (2020) ^††^ [30] U.S.	Describes the design of a stepped-wedge cluster randomized trial to evaluate the impact of an intervention that employs machine learning-based prognostic algorithms and behavioral nudges to prompt oncologists to have SICs with patients at high risk of short-term mortality.	Stepped-wedge cluster randomized controlled trial. Oncology clinics.	-	-	This trial represents a novel application of machine-generated mortality predictions combined with behavioral nudges in the routine care of outpatients with cancer.
Billie and Letizia (2020) [39] U.S.	To develop, implement, and evaluate an educational program and a serious illness protocol for a case management team of nurses and social workers.	A case management quality improvement project–pre/post intervention test. Primary care clinics.	Staff = 20 Patients = 106	20 (100%) -	Serious Illness Protocol: The case managers correctly identified 92% of patients who met the established identification criteria for this project. In 91.8% of cases, the case managers conducted a SIC in adherence to the protocol. In 76% of the cases, documentation about the SIC was completed in accordance with the protocol.
Paladino et al. (2020) ^‖^ [23] U.S.	To determine the effect of the SICP on health care utilization at the end of life in oncology.	Cluster-randomized trial. Oncology.	Patients Int. = 74 (62 y) Cont. = 83 (62 y)	41 (55%) 45 (54%)	Intervention and control patients had similar end-of-life health care utilization as measured by the mean number of NQF-endorsed indicators.
Pasricha et al. (2020) [43] U.S.	To examine the feasibility, acceptability, and utility of a standardized SIC to guide communication between nonpalliative care trained providers and surrogates of critically ill, mechanically ventilated patients.	Mixed-methods quality improvement pilot study. Intensive care.	Staff = 9 Patients = 50 Surrogate = 19	-	We found that implementation of a structured communication tool in the intensive care unit was feasible and acceptable to surrogates and providers; yet, fidelity to the timing and completion was modest. The tool appeared to yield valuable information for understanding the goals, fears, and care preferences of mechanically ventilated patients.
Lakin et al. (2020) ^¶^ [28] U.S.	This study explores whether an intervention to improve conversations about patients’ goals in a primary care setting could improve the value of healthcare delivered.	Secondary analysis of a quality improvement intervention. Primary care.	Patients = 84 (83.1 y)	47 (56%)	Possible savings observed in this study are similar in magnitude to previous studies in advance care planning and specialty palliative care but occur earlier in the disease course and in the context of documented conversations and a comprehensive, interprofessional case management program.
Paladino et al. (2020) ^‖^ [22] U.S.	This analysis evaluates the patient and clinician experience of a conversation using a SICG.	Secondary analysis from a cluster-randomized clinical trial. Oncology.	Staff = 54 Patients = 163	-	Conversations using a SICG were feasible, acceptable, and associated with positive experiences for both patients and clinicians in oncology in ways that align with national recommendations for serious illness communication.
Lakin et al. (2020) [12] U.S. and U.K.	To describe the strategies used by a collection of healthcare systems to apply different methods of identifying seriously ill patients for a targeted palliative care intervention to improve communication around goals and values.	Implementation case series. Variety of settings in 5 healthcare systems.	-	-	Involving clinical and program staff to choose a simple initial method for patient identification is the ideal starting place for selecting patients for palliative care interventions. However, improving and refining methods over time is important and we need ongoing research into better patient selection methodologies that move beyond mortality prediction and instead focus on identifying seriously ill patients—those with poor quality of life, worsening functional status, and medical care that is negatively impacting their families.
Pottash et al. (2020) [44] U.S.	To test the acceptability of incorporating a SIC into routine trainee practice.	Acceptability study, descriptive (mixed methods). Ambulatory care.	Staff = 21	5 (23%)	With preparation, time, and a conversation guide, trainees completed the elements of a SIC and found it to be an important addition to their routine practice. Patients found the conversation to be important, reassuring, and of better quality than their usual visits.
Ma et al. (2020) [42] Canada	To assess whether the quality of conversations about serious illness improved after implementation of the SICP.	Retrospective chart review study. Inpatient medical.	Staff = 21 Patients: Int. = 56 (76.2 y) Cont. = 56 (80.1 y)	30 (53.6%) 31 (55.4%)	Implementation of the SICP in a hospital setting was associated with higher quality of documented conversations regarding serious illness with patients at high risk for clinical or functional deterioration. The SICP is transferable and adaptable to a hospital setting, and was associated with an increase in adherence to best practices compared to usual care.
Wasp et al. (2020) [45] U.S.	We developed and tested an implementation strategy for incorporating the SICG into hematology-oncology fellowship training.	Prospective, single-center, cohort implementation study. Oncology.	Staff = 8	4 (50%)	Despite acquisition of communication skills, promoting new clinical behaviors remains challenging. More work is needed to identify which implementation strategies are required in this learner population.
Kumar et al. (2020) [40] U.S.	To characterize the experiences and perceptions of patients engaging in SICs as part of routine oncology care in the setting of SICP implementation.	Prospective, cross-sectional quality improvement evaluation. Oncology.	Patients = 32	17 (53%)	SICs are generally acceptable to oncology patients (non-harmful to the vast majority, positive for many). Our qualitative analysis suggests a positive impact on prognostic understanding and end-of-life planning, but opportunities for improvement in the delivery of prognosis and preparing patients for SICs.
Sanders et al. (2020) ^‖^ [24] U.S.	To describe our measurement approach to GCC, present findings from a post-hoc analysis of trial data, and discuss lessons learned about measuring GCC.	Secondary analysis of trial data. Oncology.	Patients = 203 (All) (61.8y)	106 (53%)	Measuring GCC remains a fundamental challenge to palliative care researchers. Ratings attest to the fact that many things matter to patients; however, rankings can better determine what matters most.
Gace et al. (2020) ^‡‡^ [32] U.S.	To assess patients’ experience and perception of the quality of goals and values conversations.	Two group cohort trial. Inpatient medical units.	Int. = 75 (69.8 y) Cont. = 55 (65.6 y)	40 (53.3%) 28 (50.9%)	This study suggests that informing the care team regarding their patients’ potential unmet palliative care needs is associated with patients reporting improved experience of their care without adverse effects on their mood.
Greenwald et al. (2020) ^‡‡^ [33] U.S.	To assess the impact on hospitalists of a system that reminds them to have SICs with their patients identified with potential unmet palliative needs.	Two group cohort trial. Inpatient medical units.	Staff = 61	31 (50.8%)	Routinely informing hospitalists when their patients were identified as being at increased risk for unmet palliative needs did not increase the sense of meaning these providers achieved.
Lally et al. (2020) [41] U.S.	We undertook a project to increase the number of SICs occurring in an ACO using a script delivered telephonically by nurse care managers.	Quality improvement implementation. Community care.	-	-	This project demonstrates a unique way to modify the SICG for use by nurses as part of a health care team.
Manz et al. (2020) ^††^ [31] U.S.	To determine the effect of a clinician-directed intervention integrating machine learning mortality predictions with behavioral nudges on motivating clinician-patient SICs.	Stepped-wedge cluster randomized clinical trial. Oncology clinics.	Staff = 78 Patients: Cont. = 12,170 (62.5 y) Int. = 13,889 (61.3 y)	6426 (52.8%) 7576 (54.5%)	Behavioral nudges combined with machine learning mortality predictions can positively influence clinician behavior and may be applied more broadly to improve care near the end of life.
Lakin et al. (2021) [49] U.S.	To assess the implementation of the SAGE program in a population of patients hospitalized on a general medical service.	Quality improvement implementation. Inpatient medical.	Patients:Int. = 64 (85.8 y) Comp. = 69 (85.6 y)	38 (59.4%) 46 (66.7%)	This study demonstrated significant differences in the frequency and quality of SICs completed earlier in the illness course for hospitalized patients.
Paladino et al. (2021) [51] U.S.	Describe(s) the tool development strategy, the themes that emerged from stakeholder engagement, and the two communication guides that resulted from this process.	Adaptation of SICG for COVID-19. Inpatient and outpatient COVID-19.	-	-	Well-designed communication tools and implementation strategies can equip clinicians to foster connection with patients and promote shared decision making. Although not an antidote to this crisis, such high-quality ACP may be one of the most powerful tools we have to prevent or ameliorate suffering due to COVID-19.
Paladino et al. (2021) ^¶^ [29] U.S.	To explore practical aspects of SICP implementation.	Qualitative descriptive. Primary care.	Staff = 14	10 (71.4%)	The shifts in processes employed by inter-professional clinicians revealed comprehensive models for prognostic communication and creative workflows to ensure that patients with complex illnesses had proactive, longitudinal, and patient-centered SICs and care planning.
Thamcharoen et al. (2021) [54] U.S.	This pilot study aimed to explore whether use of the SICG to aid early ACP is acceptable, and to evaluate the information gained from these conversations.	Mixed-methods implementation study. Nephrology clinic.	Patients = 26 (78y)	13 (50%)	Patients in this pilot study found the adapted SICG acceptable. This guide may be used with patients early in the course of advanced kidney disease to gather information for future ACP.
Schmidt et al. (2021) [52] U.S. and Canada	To find better methods for increasing patient recruitment for the ACP study.	Intervention study. Primary care.	Staff = 14 Patients = 120	11 (79%) -	Notifying clinical staff about potential study participants increased patient referrals in this ACP study.
Swiderski et al. (2021) [53] U.S.	(To) explore(s) the experiences of primary care physicians who participated in an initiative to implement structured SICs.	Descriptive qualitative. Community health.	Staff = 11 Patients = 37 (73y)	8 (72.7%) 37 (37.8%)	Physicians at CHCs identified challenges in SICs at personal, interpersonal, organizational, and societal levels.
Hafid et al. (2021) [47] Canada	The objective of this study was to implement ACP through adapted SICP training sessions, and to understand PCP perceptions of implementing ACP into practice.	Mixed-methods quality improvement study. Family medicine.	Staff = 34	26 (76%)	Training in ACP conversations improved PCPs’ individual perceived abilities, but discomfort and other barriers were identified.
Karim et al. (2021) [48] Canada	The aims of this initiative was to identify at least 24 patients (12 patients per clinic) for SIC and that at least 95% of all conversations would be documented in the EMR.	Implementation study. Outpatient oncology.	Staff = 2 Patients = 16 (67.8 y)	10 (62.5%)	Implementation of the SICP resulted in increased rates of documentation, but the target number of conversations was not met.
Le et al. (2021) [50] Canada	To investigate the feasibility of using the SQ to identify patients who would benefit from early SICs and study any changes in the interdisciplinary team’s beliefs, confidence, and engagement as a result of asking the SQ.	Prospective cohort pilot study. Acute medicine units.	Staff = 97 Patients: Int. = 16 Cont. = 42	-	There are ethical and practical issues as to what constitutes a ‘serious illness’ and if answering ‘no’ to the SQ always equates to a conversation. The barriers of time constraints and lack of training call for institutional change in order to prioritize the moral obligation of SICs.
DeCourcey et al. (2021) [46] U.S.	To develop a generalizable ACP intervention for children, adolescents, and young adults with serious illness using a multistage, stakeholder-driven approach.	Intervention development and adaptation. Inpatient-pediatrics.	Staff = 34 Parents = 9 Patients = 7	26 (76.5%) 4 (44.4%) 5 (71.4%)	The finalized PediSICP intervention includes a structured HCP and family ACP communication occasion supported by a 3-part communication tool and bolstered by focused HCP training. We also identified strategies to ameliorate implementation barriers.

* Age reported as mean (in years); ^†^ All data originated from, or was adapted from, the associated source indicated in the table; ^‡^ Dana-Farber Neuro-oncology Pilot Cluster; ^‖^ Dana-Farber Cluster Randomized Trial Cluster; ^¶^ Brigham Primary Care Integrated Care Management Program Cluster; ^††^ University of Pennsylvania Machine Learning Cluster Randomized Trial; ^‡‡^ Massachusetts General Hospital Cluster. Abbreviations: ACO—Accountable Care Organization; ACP—Advance Care Planning; CHC—Community Health Centre; Cont.—Control; EMR—Electronic Medical Record; GBM—Glioblastoma Multiforme; GCC—Goal Concordant Care; HCP—Health Care Professional; HF—Heart Failure; Int.—Intervention; LTACH—Long-Term Acute Care Hospital; NSQ—Nurse Surprise Question; NQF—National Quality Forum; PCP—Primary Care Provider; PSQ—Physician Surprise Question; SAGE—Speaking About Goals and Expectations; SIC—Serious Illness Conversation; SICG—Serious Illness Conversation Guide; SICP—Serious Illness Care Program; SQ—Surprise Question.

## References

[1] Bernacki R.E., Block S.D. (2014). Communication About Serious Illness Care Goals: A review and synthesis of best practices. JAMA Intern. Med..

[2] Kelley A.S., Bollens-Lund E. (2018). Identifying the Population with Serious Illness: The “Denominator” Challenge. J. Palliat. Med..

[3] Bernacki R., Paladino J., Neville B.A., Hutchings M., Kavanagh J., Geerse O., Lakin J., Sanders J.J., Miller K., Lipsitz S. (2019). Effect of the Serious Illness Care Program in Outpatient Oncology: A cluster randomized clinical trial. JAMA Intern. Med..

[4] Geerse O., Lamas D.J., Sanders J.J., Paladino J., Kavanagh J., Henrich N.J., Berendsen A.J., Hiltermann T.J., Fromme E.K., Bernacki R.E. (2019). A Qualitative Study of Serious Illness Conversations in Patients with Advanced Cancer. J. Palliat. Med..

[5] Paladino J., Bernacki R., Neville B.A., Kavanagh J., Miranda S.P., Palmor M., Lakin J., Desai M., Lamas D., Sanders J.J. (2019). Evaluating an intervention to improve communication between oncology clinicians and patients with life-limiting cancer: A cluster randomized clinical trial of the serious illness care program. JAMA Oncol..

[6] Bernacki R., Hutchings M., Vick J., Smith G., Paladino J., Lipsitz S., Gawande A.A., Block S.D. (2015). Development of the Serious Illness Care Program: A randomised controlled trial of a palliative care communication intervention. BMJ Open.

[7] Brighton L., Bristowe K.R. (2016). Communication in palliative care: Talking about the end of life, before the end of life. Postgrad. Med. J..

[8] Ariadne Labs Serious Illness Care. https://www.ariadnelabs.org/serious-illness-care/.

[9] Paladino J., Sanders J., Kilpatrick L.B., Prabhakar R., Kumar P., O’Connor N., Durieux B., Fromme E.K., Benjamin E., Mitchell S. (2022). Serious illness care programme-contextual factors and implementation strategies: A qualitative study. BMJ Support. Palliat. Care.

[10] Jacobsen J., Bernacki R., Paladino J. (2022). Shifting to Serious Illness Communication. JAMA.

[11] Smith G.M., Radigan N.J., Maloney F.L., Hawrusik R., Paquette E., Takahashi K., Downey N., Paladino J., Bernacki R.E. (2021). Development, Implementation, and Outcomes of a Serious Illness Care Community of Practice. J. Pain Symptom Manag..

[12] Lakin J.R., Desai M., Engelman K., O’Connor N., Teuteberg W.G., Coackley A., Kilpatrick L.B., Gawande A., Fromme E.K. (2019). Earlier identification of seriously ill patients: An implementation case series. BMJ Support. Palliat. Care.

[13] Lakin J., Block S.D., Billings J.A., Koritsanszky L.A., Cunningham R., Wichmann L., Harvey D., Lamey J., Bernacki R.E. (2016). Improving Communication About Serious Illness in Primary Care: A Review. JAMA Intern. Med..

[14] Arksey H., O’Malley L. (2005). Scoping studies: Towards a methodological framework. Int. J. Soc. Res. Methodol..

[15] Colquhoun H.L., Levac D., O’Brien K.K., Straus S., Tricco A.C., Perrier L., Kastner M., Moher D. (2014). Scoping reviews: Time for clarity in definition, methods, and reporting. J. Clin. Epidemiol..

[16] Levac D., Colquhoun H., O’Brien K.K. (2010). Scoping studies: Advancing the methodology. Implement. Sci..

[17] Khalil H., Peters M.D., Tricco A.C., Pollock D., Alexander L., McInerney P., Godfrey C.M., Munn Z. (2021). Conducting high quality scoping reviews-challenges and solutions. J. Clin. Epidemiol..

[18] Pollock D., Davies E.L., Peters M.D.J., Tricco A.C., Alexander L., McInerney P., Godfrey C.M., Khalil H., Munn Z. (2021). Undertaking a scoping review: A practical guide for nursing and midwifery students, clinicians, researchers, and academics. J. Adv. Nurs..

[19] Peters M.D., Marnie C., Tricco A.C., Pollock D., Munn Z., Alexander L., McInerney P., Godfrey C.M., Khalil H. (2020). Updated methodological guidance for the conduct of scoping reviews. JBI Évid. Synth..

[20] Tricco A.C., Lillie E., Zarin W., O’Brien K.K., Colquhoun H., Levac D., Moher D., Peters M.D.J., Horsley T., Weeks L. (2018). PRISMA Extension for Scoping Reviews (PRISMA-ScR): Checklist and Explanation. Ann. Intern. Med..

[21] Miranda S.P., Bernacki R.E., Paladino J.M., Norden A.D., Kavanagh J.E., Palmor M.C., Block S.D. (2017). A Descriptive Analysis of End-of-Life Conversations With Long-Term Glioblastoma Survivors. Am. J. Hosp. Palliat. Med..

[22] Paladino J., Koritsanszky L., Nisotel L., Neville B.A., Miller K., Sanders J., Benjamin E., Fromme E., Block S., Bernacki R. (2020). Patient and clinician experience of a serious illness conversation guide in oncology: A descriptive analysis. Cancer Med..

[23] Paladino J., Koritsanszky L., Neal B.J., Lakin J.R., Kavanagh J., Lipsitz S., Fromme E.K., Sanders J., Benjamin E., Block S. (2020). Effect of the Serious Illness Care Program on Health Care Utilization at the End of Life for Patients with Cancer. J. Palliat. Med..

[24] Sanders J.J., Miller K., Desai M., Geerse O.P., Paladino J., Kavanagh J., Lakin J., Neville B.A., Block S.D., Fromme E.K. (2020). Measuring Goal-Concordant Care: Results and Reflections From Secondary Analysis of a Trial to Improve Serious Illness Communication. J. Pain Symptom Manag..

[25] Lakin J.R., Koritsanszky L.A., Cunningham R., Maloney F.L., Neal B.J., Paladino J., Palmor M.C., Vogeli C., Ferris T.G., Block S.D. (2017). A Systematic Intervention to Improve Serious Illness Communication in Primary Care. Health Aff..

[26] Lakin J.R., Robinson M.G., Obermeyer Z., Powers B.W., Block S.D., Cunningham R., Tumblin J.M., Vogeli C., Bernacki R.E. (2019). Prioritizing Primary Care Patients for a Communication Intervention Using the “Surprise Questio”: A Prospective Cohort Study. J. Gen. Intern. Med..

[27] Lakin J., Benotti E., Paladino J., Henrich N., Sanders J. (2019). Interprofessional Work in Serious Illness Communication in Primary Care: A Qualitative Study. J. Palliat. Med..

[28] Lakin J.R., Neal B.J., Maloney F.L., Paladino J., Vogeli C., Tumblin J., Vienneau M., Fromme E., Cunningham R., Block S.D. (2020). A systematic intervention to improve serious illness communication in primary care: Effect on expenses at the end of life. Healthcare.

[29] Paladino J., Brannen E., Benotti E., Henrich N., Ritchie C., Sanders J., Lakin J.R. (2021). Implementing Serious Illness Communication Processes in Primary Care: A Qualitative Study. Am. J. Hosp. Palliat. Med..

[30] Manz C.R., Parikh R.B., Evans C.N., Chivers C., Regli S.H., Bekelman J.E., Small D., Rareshide C.A., O’Connor N., Schuchter L.M. (2020). Integrating machine-generated mortality estimates and behavioral nudges to promote serious illness conversations for cancer patients: Design and methods for a stepped-wedge cluster randomized controlled trial. Contemp. Clin. Trials.

[31] Manz C.R., Parikh R.B., Small D.S., Evans C.N., Chivers C., Regli S.H., Hanson C.W., Bekelman J.E., Rareshide C.A.L., O’Connor N. (2020). Effect of Integrating Machine Learning Mortality Estimates With Behavioral Nudges to Clinicians on Serious Illness Conversations Among Patients: A Stepped-Wedge cluster Randomized Clinical Trial with Cancer. JAMA Oncol..

[32] Gace D., Sommer R.K., Daubman B.-R., Greer J.A., Jacobsen J., LaSala C., Rosenberg L.B., Greenwald J.L. (2020). Exploring Patients’ Experience with Clinicians Who Recognize Their Unmet Palliative Needs: An Inpatient Study. J. Palliat. Med..

[33] Greenwald J.L., Greer J.A., Gace D., Sommer R.K., Daubman B.-R., Rosenberg L.B., LaSala C., Jacobsen J. (2020). Implementing Automated Triggers to Identify Hospitalized Patients with Possible Unmet Palliative Needs: Assessing the Impact of This Systems Approach on Clinicians. J. Palliat. Med..

[34] Lamas D.J., Owens R.L., Nace R.N., Massaro A.F., Pertsch N.J., Moore S.T., Bernacki R.E., Block S.D. (2017). Conversations About Goals and Values Are Feasible and Acceptable in Long-Term Acute Care Hospitals: A Pilot Study. J. Palliat. Med..

[35] Massmann J.A., Revier S.S., Ponto J. (2019). Implementing the Serious Illness Care Program in Primary Care. J. Hosp. Palliat. Nurs..

[36] Mandel E.I., Bernacki R.E., Block S.D. (2016). Serious Illness Conversations in ESRD. Clin. J. Am. Soc. Nephrol..

[37] O’Donnell A.E., Schaefer K.G., Stevenson L.W., DeVoe K., Walsh K., Mehra M.R., Desai A.S. (2018). Social Worker-Aided Palliative Care Intervention in High-risk Patients with Heart Failure (SWAP-HF): A pilot Randomized Clinical Trial. JAMA Cardiol..

[38] Totten A.M., Fagnan L.J., Dorr D., Michaels L.C., Izumi S., Combe A., Légaré F. (2019). Protocol for a Cluster Randomized Trial Comparing Team-Based to Clinician-Focused Implementation of Advance Care Planning in Primary Care. J. Palliat. Med..

[39] Billie M.E., Letizia M. (2020). Serious illness conversations: A case management quality improvement project. Prof. Case Manag..

[40] Kumar P., Wixon-Genack J., Kavanagh J., Sanders J.J., Paladino J., O’Connor N.R. (2020). Serious Illness Conversations With Outpatient Oncology Clinicians: Understanding the Patient Experience. JCO Oncol. Pract..

[41] Lally K., Fulton A.T., Ducharme C., Scott R., Filpo J. (2020). Using Nurse Care Managers Trained in the Serious Illness Conversation Guide to Increase Goals-of-Care Conversations in an Accountable Care Organization. J. Palliat. Med..

[42] Ma C., Riehm L.E., Bernacki R., Paladino J., You J.J. (2020). Quality of clinicians’ conversations with patients and families before and after implementation of the Serious Illness Care Program in a hospital setting: A retrospective chart review study. CMAJ Open.

[43] Pasricha V., Gorman D., Laothamatas K., Bhardwaj A., Ganta N., Mikkelsen M.E. (2020). Use of the Serious Illness Conversation Guide to Improve Communication with Surrogates of Critically Ill Patients. A Pilot Study. ATS Sch..

[44] Pottash M., Joseph L., Rhodes G. (2020). Practicing Serious Illness Conversations in Graduate Medical Education. Med. Sci. Educ..

[45] Wasp G.T., Cullinan A.M., Chamberlin M.D., Hayes C., Barnato A.E., Vergo M.T. (2020). Implementation and Impact of a Serious Illness Communication Training for Hematology-Oncology Fellows. J. Cancer Educ..

[46] DeCourcey D.D., Partin L., Revette A., Bernacki R., Wolfe J. (2021). Development of a Stakeholder Driven Serious Illness Communication Program for Advance Care Planning in Children, Adolescents, and Young Adults with Serious Illness. J. Pediatr..

[47] Hafid A., Howard M., Guenter D., Elston D., Fikree S., Gallagher E., Winemaker S., Waters H. (2021). Advance care planning conversations in primary care: A quality improvement project using the Serious Illness Care Program. BMC Palliat. Care.

[48] Karim S., Lupichuk S., Tan A., Sinnarajah A., Simon J. (2021). Real World Implementation of the Serious Illness Care Program in Cancer Care: Results of a Quality Improvement Initiative. J. Palliat. Med..

[49] Lakin J.R., Arnold C.G., Catzen H.Z., Rangarajan A., Berger R.S., Brannen E.N., Cunningham R.J., Schaffer A.C., Lamey J., Baker O. (2021). Early serious illness communication in hospitalized patients: A study of the implementation of the Speaking About Goals and Expectations (SAGE) program. Healthcare.

[50] Le K., Lee J., Desai S., Ho A., van Heukelom H. (2021). The Surprise Question and Serious Illness Conversations: A pilot study. Nurs. Ethics.

[51] Paladino J., Mitchell S., Mohta N., Lakin J.R., Downey N., Fromme E.K., Gullo S., Benjamin E., Sanders J.J. (2021). Communication Tools to Support Advance Care Planning and Hospital Care During the COVID-19 Pandemic: A Design Process. Jt. Comm. J. Qual. Patient Saf..

[52] Schmidt M.E., Daly J.M., Xu Y., Levy B.T. (2021). Improving Iowa Research Network Patient Recruitment for an Advance Care Planning Study. J. Prim. Care Community Health.

[53] Swiderski D., Georgia A., Chuang E., Stark A., Sanders J., Flattau A. (2021). “I was not able to keep myself away from tending to her immediate needs”: Primary Care Physicians’ Perspectives of Serious Illness Conversations at Community Health Centers. J. Gen. Intern. Med..

[54] Thamcharoen N., Nissaisorakarn P., Cohen R.A., Schonberg M.A. (2021). Serious Illness Conversations in advanced kidney disease: A mixed-methods implementation study. BMJ Support. Palliat. Care.

[55] Downar J., Goldman R., Pinto R., Englesakis M., Adhikari N.K. (2017). The “surprise question” for predicting death in seriously ill patients: A systematic review and meta-analysis. Can. Med. Assoc. J..

[56] White N., Kupeli N., Vickerstaff V., Stone P. (2017). How accurate is the ‘Surprise Question’ at identifying patients at the end of life? A systematic review and meta-analysis. BMC Med..

[57] Gonzalez-Jaramillo V., Ochoa L.F.A., Saldarriaga C., Krikorian A., Vargas J.J., Gonzalez-Jaramillo N., Eychmüller S., Maessen M. (2021). The ‘Surprise question’; in heart failure: A prospective cohort study. BMJ Support. Palliat. Care.

[58] Ernecoff N.C., Abdel-Kader K., Cai M., Yabes J., Shah N., Schell J.O., Jhamb M. (2021). Implementation of Surprise Question Assessments using the Electronic Health Record in Older Adults with Advanced CKD. Kidney360.

[59] Masso M., Allingham S., Banfield M., Johnson C., Pidgeon T., Yates P., Eagar K. (2015). Palliative Care Phase: Inter-rater reliability and acceptability in a national study. Palliat. Med..

[60] Mather H., Guo P., Firth A., Davies J., Sykes N., Landon A., Murtagh F. (2017). Phase of Illness in palliative care: Cross-sectional analysis of clinical data from community, hospital and hospice patients. Palliat. Med..

[61] Billings J.A., Bernacki R. (2014). Strategic Targeting of Advance Care Planning Interventions: The goldilocks phenomenon. JAMA Intern. Med..

[62] Flierman I., Nugteren I.C., van Seben R., Buurman B.M., Willems D.L. (2019). How do hospital-based nurses and physicians identify the palliative phase in their patients and what difficulties exist? A qualitative interview study. BMC Palliat. Care.

[63] Izumi S., Fromme E.K. (2017). A Model to Promote Clinicians’ Understanding of the Continuum of Advance Care Planning. J. Palliat. Med..

